# Respiratory Muscle Strength as an Indicator of the Severity of the Apnea-Hypopnea Index: Stepping Towards the Distinction Between Sleep Apnea and Breath Holding

**DOI:** 10.7759/cureus.14015

**Published:** 2021-03-21

**Authors:** Vasileios T Stavrou, Kyriaki Astara, Eleni Karetsi, Zoe Daniil, Konstantinos I Gourgoulianis

**Affiliations:** 1 Laboratory of Cardio-Pulmonary Testing and Pulmonary Rehabilitation, Respiratory Medicine Department, Faculty of Medicine, University of Thessaly, Larissa, GRC

**Keywords:** respiratory muscle strength, sleep apnea, male, cardiopulmonary function

## Abstract

Background and objective

The aim of this study was to investigate whether the maximum inspiratory and expiratory pressure are correlated with the apnea-hypopnea index (AHI) in patients with obstructive sleep apnea syndrome (OSAS).

Methods

Fifty-two patients with OSAS were divided into two groups (AHI, events/hours: <30, n=28, versus ≥30, n=24). For each patient, anthropometric characteristics, spirometry parameters, maximum inspiratory (MIP) and expiratory pressure (MEP), and cardiopulmonary function (CPF) parameters (oxygen uptake at rest (VO_2_), carbon dioxide output (VCO_2_), heart rate (HR), minute ventilation (V_E_), tidal volume at inspiratory (TVin) and expiratory (TVex), breath frequency (*f*_β_), end-tidal carbon dioxide pressure (P_ET_CO_2_), end-tidal oxygen pressure (P_ET_O_2_), and mean arterial pressure (MAP)) in sitting position for three minutes were recorded. The independent t-test was used to measure the differences between groups (events/hours <30 versus ≥30) and Pearson correlation analysis was used for statistical comparison between parameters.

Results

Results showed differences between groups (AHI, events/h ≥30 versus <30) in MIP (102.0±18.3 versus 91.1±12.1 % of predicted, p=0.013) and CPF parameters TVin (0.8±0.2 versus 0.7±0.1, L, p=0.047), P_ET_CO_2_ (34.6±4.2 versus 31.4±3.7, mmHg, p=0.007), and MAP (88.4±6.5 versus 82.9±6.2, mmHg, p=0.003). Pearson correlation analysis between respiratory muscle strength (MIP and MEP) and polysomnography (PSG) parameters, MIP is related to AHI (r=.332, p=0.016) and desaturation index (r=.439, p=0.001), as well as MEP to percent of REM sleep stage (r=-.564, p<0.001).

Conclusion

The data from the present study support that maximal inspiratory pressure relates to the severity of AHI and intermittent breath-holding during sleep increases the inspiratory muscle strength.

## Introduction

Obstructive sleep apnea syndrome (OSAS) is a common condition affecting 9% to 38% of the general population [1] and is characterized by recurrent upper airway collapse during sleep, leading to intermittent nocturnal hypoxia and sleep fragmentation and resulting in major pathophysiological changes [2]. OSAS symptomatology appears as a reduction (hypopnea) or complete cessation (apnea) of airflow through the airways despite continued respiratory efforts and is diagnosed by clinical history and polysomnography (PSG) [2]. According to Mendes et al., OSAS is classified by an apnea-hypopnea index (AHI >15 or an AHI >5) with daytime and nighttime symptoms while apnea severity is classified as mild (AHI 5 to 15), moderate (AHI 15.01 to 30), or severe (AHI>30.1) [3]. Patients with OSAS experience increased resistive load as compared to normal subjects while intermittent hypoxemia and sleep deprivation or fragmentation impair inspiratory muscle endurance and lower respiratory function [4].

On the other hand, breath-holding (BH) is a widespread tactic of athletes to improve endurance by weakening the chemical signals of hypercapnia that trigger breathing [5]. The difference with OSAS is that the suppression of breathing occurs voluntarily by maintaining respiratory muscles at a chosen volume, along with involuntary diaphragmatic breathing movements [6]. Hence, during a maximal inspiratory BH, the pulmonary stretch receptors are activated, sending central signals to ameliorate the urge to breath [7]. This protective stimulus during respiratory distress in OSAS, to restore breathing, seems to be attenuated [2].

The purpose of this study was to investigate whether the maximum inspiratory and expiratory pressure are associated with the apnea-hypopnea index in patients with OSAS. Moreover, we examined possible differences among patients in cardiopulmonary function in resting relate to the severity of the syndrome. We hypothesized that the OSAS syndrome could affect the respiratory muscle strength independent of the severity of the syndrome.

## Materials and methods

Participants

Fifty-two newly diagnosed male volunteers patients with OSAS were consecutively enrolled in our study and divided into two groups (AHI: <30 events/hours, n=28, versus AHI: ≥30 events/hours, n=24). All subjects completed the PSG study in the Sleep Apnea Laboratory of Respiratory Medicine Department, University of Thessaly [8]. Exclusion criteria were age <20 and >60 years old, comorbidity, body mass index (BMI) ≥40 kg/m^2^, neurological and psychiatric disorders, musculoskeletal disorders [9], daily physical activity [10], manual work, and weekly exercise ≥100 min [11].

Data collected

For each patient, anthropometric and morphological characteristics (body height, body mass, body mass index (BMI = weight (kg)/height (m)2), neck, waist and hip circumference, and body composition (body fat and total body water, Tanita MC-980: Tanita Corporation of America, Inc., Arlington Heights, Illinois)) were recorded. Moreover, calculating the body surface area (BSA = (height (cm) x weight (kg))/3600½) and estimated the lean body mass (LBM (kg) = 0.407 x weight (kg) + 0.267 x height (cm)-19.2).

All participants underwent standard spirometry and lung volume measurements, in line with American Thoracic Society (ATS)/European Respiratory Society (ERS) guidelines [12]. For each pulmonary function test, three maximal flow-volume loops were obtained to determine forced vital capacity (FVC) and forced expiratory volume in the first second (FEV1) [2].

In addition, we recorded the maximum inspiratory (MIP) and expiratory pressure (MEP) by a MicroRPM portable device (Care Fusion, California) and calculated the percent of predicted values according to the equation [13]:

MIP (cmH2O) = 142 - (1.03 x Age(yrs)), MEP (cmH2O) = 180 - (0.91 x Age (yrs))

Moreover, we recorded parameters for cardiopulmonary function at rest (oxygen uptake (VO2, ml/min), carbon dioxide output (VCO2, ml/min), heart rate (HR, bpm), minute ventilation (VE, L/min), tidal volume in inspiratory (TVin, L) and expiratory (TVex, L), breath frequency (fβ, 1/min), end-tidal carbon dioxide pressure (P_ET_CO_2_, mmHg), end-tidal oxygen pressure (P_ET_O_2_, mmHg)) in sitting position for three minutes by a MasterScreen-CPX (VIASYS HealthCare, Germany) [2]. Moreover, a 12-lead electrocardiogram (ECG) was also employed for HR monitoring while a pulse oximeter (MasterScreen, Germany) informed about SpO2. Blood pressure (cuff manometry, Mac, Japan) was recorded prior to the cardiopulmonary function assessment [2].

All sessions were performed in the Laboratory of Cardiopulmonary Testing and Pulmonary Rehabilitation, Respiratory Medicine Department, University of Thessaly, with the environmental temperature at 24±1 °C and humidity at 46±5%. The evaluation was made between 10:00 a.m. and 13:00 p.m.

Statistical analysis

The Kolmogorov-Smirnov test was used for the normality of the distribution. The independent t-test was used between groups (events/hours <30 versus ≥30). Pearson correlation analysis was used for statistical comparison between parameters. For each test, the level of significance was set to p<0.05. Continuous variables of interest were characterized by mean values with standard deviation (Mean±Sd). The IBM Statistical Package for the Social Sciences (SPSS) 21 statistical package (IBM Corp., Armonk, NY) was used for statistical analyses.

## Results

Polysomnography study

Results showed a difference between groups in polysomnography study parameters. Patients with AHI ≥30 events/hours observed higher values compare to the group with AHI <30 events/hours in apnea, desaturation index, and minimum oxygen saturation during sleep (Table 1). The percentage distribution of the stages of sleep the group with AHI ≥30 events/hours showed higher values in Stage 1 (4.7±2.7 versus 3.0±1.6, %, t(46)=2.695, p=0.010) and lower values in Stage 3-4 (8.4±5.1 versus 19.3±5.6, %, t(50)=-7.247, p<0.001) compared to the group with AHI <30 events/hours. Stage 2 (AHI ≥30 events/hours: 59.5±7.6 versus AHI <30 events/hours: 60.9±13.1, %, t(50)=0.500, p=0.619) and REM stage (AHI ≥30 events/hours: 12.1±6.1 versus AHI <30 events/hours: 11.5±4.9, %, t(50)=-0.354, p=0.725) were not different between the groups. The parameters Epworth sleepiness scale (ESS) questionnaire and sleep duration during PSG and hypopnea were not different between groups (Table 1).

**Table 1:** Results between patients with OSAS Continuous variables are presented as mean ± standard deviation. Note: OSAS = obstructive sleep apnea syndrome; AHI = apnea-hypopnea index; FEV1 = forced expiratory volume in the first second; FVC = forced vital capacity; minimum SaO2 = minimum oxygen saturation during sleep

	Apnea-Hypopnea Index		P-value
	<30 events/h	≥30 events/h	
Age, years	46.9 ± 10.2	43.8 ± 10.7	0.289
Body Mass Index, kg/m^2^	30.0 ± 4.0	32.5 ± 5.0	0.052
Body Surface Area, m^2^	2.3 ± 0.6	2.4 ± 0.5	0.654
Lean Body Mass, kg	65.9 ± 9.9	67.4 ± 9.1	0.586
Total Body Water, %	52.7 ± 2.7	51.6 ± 2.8	0.218
Body Fat, %	30.6 ± 4.5	28.6 ± 4.3	0.327
Neck Circumference, cm	40.9 ± 4.1	40.7 ± 4.5	0.921
Waist Hip Ratio, cm	1.0 ± 0.1	1.0 ± 0.1	0.818
AHI, Events/h	21.9 ± 3.8	59.3 ± 19.7	<0.001
Apnea, Events/h	4.1 ± 3.4	36.1±24.7	<0.001
Hypopnea, Events/h	23.6±14.1	23.4±12.6	0.958
Epworth Sleepiness Scale, score	8.9 ± 2.9	7.8 ± 4.6	0.295
Desaturation Index, %	18.6±5.7	60.6±21.9	<0.001
minSaO_2_, %	82.4± 6.1	77.4 ± 11.0	0.043
Sleep Duration, min	321.8± 62.0	295.2 ± 65.4	0.121
FEV_1_, % pred	111.6±37.3	99.1±11.6	0.189
FVC, % pred	118.5±37.8	102.3±15.2	0.054

Spirometry and respiratory strength

**Figure 1:**
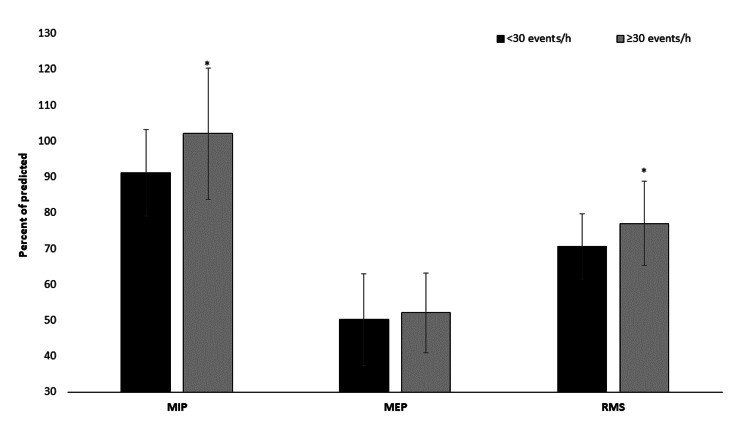
Results between groups in maximum inspiratory (MIP), expiratory pressure (MEP), and respiratory muscle strength (RMS = MIP - MEP ratio) *p<0.05

Additionally, MIP showed differences between groups. Patients with AHI ≥30 events/hours observed higher values as compared to the group with AHI <30 events/hours (102.0±18.3 versus 91.1±12.1 % of predicted, t(50)=2.590, p=0.013, Figure 1). The MEP (AHI ≥30 events/hours: 52.1±11.1 versus AHI <30 events/hours: 50.1±12.8, % of predicted, t(50)=0.579, p=0.565, Figure 1), FEV1 (AHI ≥30 events/hours: 99.1±11.6 versus AHI <30 events/hours: 111.6±37.3, % of predicted, t(50)=-0.613, p=0.189) and FVC (AHI ≥30 events/hours: 102.3±15.2 versus AHI <30 events/hours: 118.5±37.8, % of predicted, t(50)=-1.971, p=0.054) were not different between groups (Table 1).

Cardiopulmonary function

Results showed a difference between groups in cardiopulmonary function parameters. Patients with AHI ≥30 events/hours observed higher values compared to the group with AHI <30 events/hours in tidal volume during inspiration (TVin: 0.8±0.2 versus 0.7±0.1, L, t(50)=2.041, p=0.047), end-tidal carbon dioxide pressure (P_ET_CO_2_: 34.6±4.2 versus 31.4±3.7, mmHg, t(50)=2.838, p=0.007), and mean arterial pressure (MAP: 88.4±6.5 versus 82.9±6.2, mmHg, t(50)=3.175, p=0.003) (Table 2). The parameters oxygen uptake, carbon dioxide output, heart rate, ventilation, tidal volume in expiratory, breath frequency, and end-tidal oxygen pressure were not different between groups (Table 2).

**Table 2:** Cardiopulmonary function results Continuous variables are presented as mean ± standard deviation. Note: fβ = breath frequency; HR = heart rate; MAP = mean arterial pressure; P_ET_CO_2_ = end-tidal carbon dioxide pressure; P_ET_O_2_ = end-tidal oxygen pressure; VCO2 = carbon dioxide output, VE = minute ventilation; VO2 = oxygen uptake

	Apnea-Hypopnea Index		P-value
	<30 events/hours	≥30 events/hours	
VO_2_, ml/min	330.7±10.3	348.9±72.5	0.472
VCO_2_, ml/min	249.1±97.1	278.9±66.5	0.211
V_E_, L/min	9.9±3.7	11.2±2.0	0.128
Tidal Volume inspiratory, L	0.7±0.1	0.8±0.2	0.047
Tidal Volume expiratory, L	0.6±0.2	0.7±0.2	0.062
f_β_, 1/min	15.7±3.8	16.3±4.1	0.626
P_ET_CO_2_, mmHg	31.4±3.7	34.6±4.2	0.007
P_ET_O_2_, mmHg	109.8±4.5	109.5±6.1	0.826
HR, bpm	77.6±6.6	84.1±13.7	0.030
MAP, mmHg	82.9±6.2	88.4±6.5	0.003

Patient’s characteristics

Anthropometric and morphological characteristics weren’t different between groups (Table 1).

Correlation results

According to Spearman's correlation analysis between respiratory muscle strength (MIP and MEP) and PSG parameters, MIP is related to AHI (r= .332, p=0.016) and desaturation index (r= .439, p=0.001) and MEP is related to percent of REM sleep stage (r= -.564, p<0.001) and sleep duration (r= -.309, p=0.026).

## Discussion

The aim of this study was to investigate whether the maximum inspiratory and expiratory pressure are correlated with the apnea-hypopnea index in patients with OSAS. Our main finding was that MIP is related to the severity of AHI and affected by the desaturation index.

Previous studies have reported that AHI severity was correlated with some respiratory parameters, as well as mean arterial pressure [11]. The increase of tidal volume during inspiration and of P_ET_CO_2_ is indicative of the instability of central respiratory motor output to airway and pump muscles during sleep while the association between OSAS and hypertension has been well-established in the context of chronic sympathetic excitation [10]. In the present study, we observed higher values in P_ET_CO_2_, tidal volume in inspiratory, mean arterial pressure, and heart rate in resting in patients with AHI ≥30 compared to the group with <30 events/hours. In agreement with our findings, Stavrou et al. reported increased P_ET_CO_2_ (approximately 4 mmHg) in patients with OSAS as compared to the control group [2].

Our data present that patients with AHI ≥30 events/hours had higher values in TV in inspiratory compared to patients with AHI <30 events/hours group (Table 2). Breathing is the exchange of O_2_ and CO_2_ gases in order to remove the excess of CO_2_ from the system. CO_2_ is a regulator of blood pH and its fluctuations may disturb the homeostasis if levels are not maintained in the normal range [2]. Breathing occurs automatically and is regulated, according to the metabolic demands, by the autonomic nervous system (ANS) and, more specifically, by the interconnection of the vital reflexes (respiratory (RC), vasomotor (VMC), and cardiac centers (CC)) located in the medulla oblongata, with the RC being the principal regulator [14]. Voluntary control of breathing and, therefore, the involvement of distinct areas of the cerebral cortex is possible but only temporally [6]. In our data, patients with AHI ≥30 events/hours had higher values P_ET_CO_2_ at 3.1 mmHg than patients with AHI <30 events/hours group. The increased P_ET_CO_2_, like the ones measured in our patients, is an end product of a complex conglomerate influenced by factors such as the severity of sleep apnea, daytime PaO2, blunted respiratory drive, respiratory mechanics, and respiratory muscle fatigue [2]. If apnea is willingly prolonged - as seen in professional athletes to improve endurance - various physiological processes occur to compensate for the detrimental effects of CO_2_ retention. Central and peripheral chemoreflexes are activated due to hypoxia, the sympathetic nervous system (SNS) tone is increased due to the stimulation of VMC and CC, leading to hypertension and bradycardia due to the stimulation of the vagus nerve, and lung volume is reduced due to chest compression and dilation of thoracic vasculature [15]. However, the effects are transient and reversible during rest time intervals.

According to our results, observed higher values in MIP in patients with AHI ≥30 events/hours as compared to the <30 events/hours group. The intermittent breath-holding during hypoxia re-oxygenation in patients with OSAS probably increases the intrathoracic pressure with successive alteration in the transmural pressure of the cardiac cavities, similar to the ones produced during exercise [16]. During the repeated process of hypoxia re-oxygenation, the respiratory muscles increase their fatigue strength [17], which leads to major immediate pathophysiological changes of breath [18]. In the present study, we observed a positive relationship between MIP and AHI (r= .332, p=0.016) and desaturation index (r= .439, p=0.001).

Regulation of breathing during sleep is principally under the control of chemoreceptors while many of the inputs witnessed in wakefulness and, therefore, BH, are diminished [19]. Consequently, the ventilatory feedback control system of chemoreflex is based on the fluctuations of PaO2, which are more prominent during OSAS, making it vulnerable to instability [20]. OSAS consists of repetitive episodes of apneas and hypopneas, which activate the circle intermittent hypoxia-hypercapnia [21], results that are compatible with our findings (P_ET_CO_2_: 31.4±3.7 versus 34.6±4.2 mmHg). The chemoreflex is stimulated entirely, increasing the SNS tone during both sleep and wakefulness, leading to the clinical manifestation of hypertension and tachycardia [22] in contrast to BH in trained breath-hold divers to whom PNS is not attenuated to OSAS’ extent. The present study observed higher values in HR at rest in patients with AHI ≥30 events/hours as compared to the <30 events/hours group (77.6±6.6 versus 84.1±13.7, bpm). In fact, BH divers probably develop enhanced and impermanent chemo-responsiveness to hypoxia with a blunted ventilatory response to hypercapnia [23]. Therefore, it could be suggested that chemoreflex abnormalities in OSAS cannot be merely explained by intermittent arterial oxygen desaturation [24]. Besides, sympathoexcitation occurs in elite divers too, without the detrimental effects of chronic intermittent hypoxia witnessed in OSAS, posing a challenge in truly understanding the mechanisms and effects of chemoreflex upregulation [25].

The suboptimal length of a muscle such as the diaphragm leads to the suboptimal release of force according to the basic properties of muscles [26]. MIP is a measure of diaphragmatic inspiratory muscle strength, which results in being increased in OSAS, as strength weakens [27]. Respiratory muscle function has been correlated with vital capacity, indicating that respiratory muscle weakness contributes to hypoventilation [28]. Thus, there has been an interest in the literature on the beneficial effects of increasing the respiratory and especially the inspiratory muscle strength in both sports and sleep-disordered breathing (SDB). Training protocols that involve BH [5] have been proven to be effective in improving performance while inspiratory muscle training (IMT) alleviates accompanying SDB hypertension. It has been proposed that the conscious apnea stimulus is beneficial, as it resembles the effects of aerobic exercise, benefiting MIP [29]. It differs from sleep apneic episodes as, during the rest of the intervals of BH, the arterial blood gases and acid-base status are fully reversed, attenuating the increased tone of SNS that persists in OSAS [30].

Nevertheless, in our study, there were some limitations. The participants were only men and the low number of participants might be a statistical bias in our conclusions.

## Conclusions

To conclude, data from the present study support that the maximal inspiratory pressure associated with the severity of AHI and intermittent BH during sleep increases the inspiratory muscle strength. However, its effects differ, since OSAS is detrimental due to the sympathoexcitation that persists, contributing to comorbidities, whereas BH is not only reversible but also beneficial for athletic performance. As oxygen desaturation and arterial re-oxygenation seem to affect only chemoreflex upregulation in part, further research on respiratory muscle strength as a potential candidate for elucidating the relationship of apneas in OSAS and BH is required.
